# Does three-dimensional functional infrared imaging improve breast cancer detection based on digital mammography in women with dense breasts?

**DOI:** 10.1007/s00330-019-06248-y

**Published:** 2019-05-21

**Authors:** Roxanna J. Hellgren, Ann E. Sundbom, Kamila Czene, David Izhaky, Per Hall, Paul W. Dickman

**Affiliations:** 1 Department of Medical Imaging, Division of Breast Imaging, Södersjukhuset, 118 83 Stockholm, Sweden; 2 Department of Medical Epidemiology and Biostatistics, Karolinska Institutet, 171 77 Stockholm, Sweden; 3 Department of Research and Development, Real Imaging, 1 Golan St., 7019802 Airport City, Israel; 4 Department of Oncology, Södersjukhuset, 118 83 Stockholm, Sweden

**Keywords:** Mammography, Breast neoplasms, Breast density, Magnetic resonance imaging, Risk assessment

## Abstract

**Purpose:**

We aimed to estimate the incremental cancer detection rate achieved by adding three-dimensional functional infrared imaging (3DIRI) to digital mammography in women with dense breasts.

**Materials and methods:**

In this prospective study conducted between December 2014 and April 2016, 1727 women (median age 56) with percentage volumetric breast density > 6% were recruited at routine screening mammography to undergo additional 3DIRI. The 3DIRI findings were classified as negative or positive. Women with a negative mammography but positive 3DIRI were referred to dynamic contrast-enhanced MRI, whereas all other women underwent routine follow-up based on the mammography finding. Diagnosis of breast cancer was verified by histopathologic examination. The number of women diagnosed with a malignancy formed the basis of our statistical analysis.

**Results:**

Mammography detected 7 cancers in 7 women. Of 1692 women with negative mammography, 222 women (13%) had a positive 3DIRI of which 219 underwent MRI. An additional 6 cancers were identified in 5 women, increasing the diagnostic yield from 7 of 1727 (0.41%) to 12 of 1727 (0.69%). The incremental cancer detection rate associated with using 3DIRI to select women for MRI was 5 of 222 (22.5 additional cancers per 1000).

**Conclusion:**

The use of 3DIRI to select women for an additional MRI can result in the detection of additional cancers in women with dense breasts, but at the expense of additional false positives and considerably lower positive predictive value of the combined examinations. Additional studies are necessary to evaluate the role of 3DIRI as an adjunct to mammography.

**Key Points:**

• *Use of three-dimensional functional infrared imaging to select women for an MRI in addition to screening mammography has the potential to improve breast cancer detection in women with dense breasts.*

**Electronic supplementary material:**

The online version of this article (10.1007/s00330-019-06248-y) contains supplementary material, which is available to authorized users.

## Introduction

Mammography screening reduces breast cancer mortality, but the sensitivity of mammography declines with increasing breast density; women with extremely dense breasts have a 6 times higher odds of interval cancer compared to women with predominantly fatty breasts [1]. A number of adjunct imaging methods such as tomosynthesis [2–6], ultrasound [7–12], and MRI [13] have been implemented. All methods have both advantages and limitations and new methods deserve investigation.

In a previous proof of concept study, three-dimensional functional infrared imaging (3DIRI) showed high sensitivity in assessing the likelihood of breast cancer [14] and may be a useful addition to the arsenal of tools in breast cancer diagnosis. This method, based on physiology and metabolic changes, is non-invasive, does not involve ionizing radiation, involves no contact with the breasts, and is thought to operate independent of breast density. Unlike previous-generation thermography that only identified thermal variations between contralateral breasts, the 3DIRI device generates three-dimensional vascular maps of the breasts by combining structured-light imaging and optical imaging in the infrared spectrum. The 3DIRI data are used to detect peripheral breast vasculature asymmetry as well as variations in vascular morphology, density, and perfusion rate between the breasts. This is believed to correspond to the MRI-detected asymmetric increased breast vascularity, a biological marker for breast malignancy [15–18].

The aim of this prospective study was to assess the clinical benefit, measured by the incremental cancer detection rate, of adding 3DIRI to population-based mammography screening of asymptomatic women with dense breast tissue.

## Materials and methods

### Participants

This prospective study was performed at a single imaging center at Stockholm South General Hospital. The local ethical review board approved the study. All participating women provided written informed consent.

All women enrolled in the study were participants in the Karolinska Mammography Project for Risk Prediction of Breast Cancer (KARMA; karmastudy.org), a prospective cohort study of more than 70,000 women, initiated in January 2011 [19]. During the period December 2014–April 2016, we invited asymptomatic women who attended population-based mammography screening (ages 40–74) to participate in our study. We only invited women with a breast density in the highest two-thirds of the population, corresponding to density on their previous mammogram greater than 6% (mean of left and right breasts) according to automated Volpara volumetric breast density analysis (Volpara Solutions) [20]. Women then underwent a mammogram as part of our study. Due to variation/reduction of density, some women had a density of less than 6% on the new mammogram. Women with previous cancers, previous breast surgery, recent breast biopsy, or ongoing pregnancy were excluded from the study.

### Study design

Study participants underwent, on the same day, 3DIRI in addition to their standard care mammography screening. All women with a positive mammogram underwent clinical workup with additional mammography imaging, ultrasound, and biopsy of positive findings. All women with a negative mammogram (including cases of positive mammogram and negative clinical workup) and positive 3DIRI score were referred to undergo an MRI. The reason for choosing MRI is that it has the highest sensitivity of available imagining methods and has particularly good properties for detecting cancer in women with dense breasts [13]. Since our aim was to rule out or confirm the presence of malignancy in 3DIRI-positive women, we chose MRI due to its high sensitivity. Women with a positive MRI underwent further clinical workup and biopsy of positive findings. Women who could not undergo MRI (e.g., due to feelings of claustrophobia) underwent ultrasound examination.

### Imaging

#### 3D functional infrared imaging

We used a prototype 3DIRI system (Real Imaging Ltd), calibrated as described by Sella et al [14]. The device is further described in Appendix 1. We performed 3DIRI image acquisition prior to mammography screening to avoid artifacts that might be caused by the screening procedure (e.g., compression by mammography). Participants were imaged while sitting in an upright position and were asked to maintain this position throughout the imaging session, with emphasis on the importance of not moving or changing position. The 3DIRI software generates a score from − 100 to 100 for cancer likelihood which we then dichotomized into healthy (*negative*) or suspicious for malignancy (*positive*). The risk score pertains to both breasts and does not give information on laterality or location. The risk model was not changed throughout the study period.

#### Dynamic contrast-enhanced magnetic resonance imaging

MRI was performed according to the guidelines of the European Society of Breast Imaging [21]. In premenopausal women, MRI was performed on days 7–14 of the menstrual cycle. Participants underwent MRI in the prone position using a 1.5-Tesla MAGNETOM Aera (Siemens Medical Solutions) device with a dedicated 16-channel breast coil and a standard dynamic DCE-MRI protocol. Gadolinium contrast material (Dotarem; GE Healthcare) was administered intravenously 0.2 ml/kg as a bolus injection with injector followed by 15 ml saline solution.

#### Breast ultrasound

Ultrasound examinations were performed with iU22 vision 2010 US system (Philips Medical Systems) with a L17–5 linear array probe or a L12–5 linear array probe for large breasts.

#### Mammography

Screening mammography was performed using the Philips Microdose system (Philips Healthcare) with image acquisition in the CC and MLO view. Clinical mammography was performed using Philips Mammo Diagnost DR (Philips Medical Solutions) and Siemens MammoMat 3000 Nova (Siemens AG, Medical Solutions).

### Image interpretation

All images were interpreted in conjunction with clinical history and, when available, prior imaging. Radiologists were blinded to 3DIRI results when evaluating the mammograms, but aware of positive 3DIRI risk score when assessing MRI examinations.

Assessments were made in accordance with the national 5-point classification scale (same as the British Royal College of Radiologists Breast Group imaging classification) where 1 is normal, 2 is benign, 3 is intermediate/probably benign finding, 4 is findings suspicious of malignancy, and 5 is findings highly suspicious of malignancy [22, 23]. Images classified 1–2 were considered negative and images classified 3–5 were considered positive and required further workup.

For mammography, 5 radiologists with 6–30 years of experience were responsible for evaluation of screening mammograms. Two radiologists read each mammogram. For MRI, 3 radiologists with 3–8 years of experience in mammography/ultrasound and 0–2 years of experience in DCE-MRI were responsible for evaluation. Each MRI examination was read by 2 radiologists. When an agreement could not be reached, a radiologist with 7 years of breast MRI experience was consulted and a consensus reached. The *American College of Radiology breast imaging reporting and data system* (BIRADS) lexicon 5th edition was used for MRI image interpretation and reporting, but assessments were given according to the 5-point scale described above.

### Verification

All women with positive mammograms underwent standard workup with complimentary image acquisition and ultrasound. All positive findings were verified by needle biopsy guided by either ultrasound or stereotactic technique.

All women with a negative mammogram and positive 3DIRI were further examined by MRI (in three cases where MRI could not be performed because of patient anxiety, ultrasound was performed). MRI was considered the imaging gold standard. Women with positive MRIs underwent further mammographic and ultrasonic workup and biopsy. If the finding could not be visualized by these methods, MRI-guided vacuum-assisted biopsy was performed. Histopathology reports were retrieved to verify the diagnosis of breast cancer.

All women were followed up in the National Breast Cancer Registry to identify a diagnosis of breast cancer during a period of at least 17 months subsequent to the initial examination. Women underwent the initial examination between November 2015 and June 2016 and were followed up in the national registry until 31 October 2017.

### Statistical analysis

The number of *women* diagnosed with a malignancy formed the basis of our statistical analysis rather than, for example, the number of diagnosed tumors relative to the number of *breasts* examined. We calculated the diagnostic yield as the proportion of women with a positive biopsy among all women who participated in screening. We calculated the diagnostic yield based on both screening mammography alone and when 3DIRI was combined with screening mammography. We also calculated the incremental cancer detection rate as the number of additional cancers diagnosed among women referred for an MRI divided by the number of women referred for an MRI. Confidence intervals (95%) for proportions were calculated using Wilson’s approximation [24]. We chose to present our results primarily as a schematic diagram showing the number of women at each step of the study, number of positive and negative tests, number of biopsies, and number of diagnosed cancers. A number of statistics can be calculated based on these data, but we chose to present the diagnostic yield (incremental cancer detection rate) and positive predictive value of biopsy since these are the most relevant statistics for our primary aim.

## Results

### Participant characteristics

A total of 1804 asymptomatic women were enrolled and underwent 3DIRI and screening mammography at Stockholm South General Hospital between November 2015 and June 2016. Of these 1804 women, 39 (2%) were excluded due to 3DIRI device malfunction and 38 (2%) were excluded due to protocol deviations (participant withdrawal, participant recalled additional information (e.g., breast surgery) after enrollment that necessitated exclusion, mammography performed more than 7 days after 3DIRI), leaving 1727 (96%) women eligible for the study. A description of the study population is shown in Table 1. The median age of the study participants was 56 years (age range 43–74 years). Approximately 89% of women had volumetric breast density greater than 7.5%, which is considered dense [20].

**Table 1:** Characteristics of study participants, number (%)

Age at screening	
40–49	358 (21%)
50–59	707 (41%)
60–69	461 (27%)
70–74	201 (12%)
Volpara breast density measurement	
< 4.5%	10 (1%)
4.5–7.5%	185 (11%)
7.5–15.5%	1173 (68%)
> 15.5%	359 (21%)
Menopausal status	
Pre-/peri-menopausal	596 (35%)
Post-menopausal	1131 (65%)
BMI	
< 20	157 (9%)
20–24.9	1150 (67%)
25–30	360 (21%)
> 30	54 (3%)
Risk factor	
Known mutation for BRCA1/BRCA2	1
Family history of breast cancer	234 (14%)

Numbers in parentheses are percentages, which are rounded. As such, some percentages may not sum to 100

### Cancer detection

**Fig. 1:**
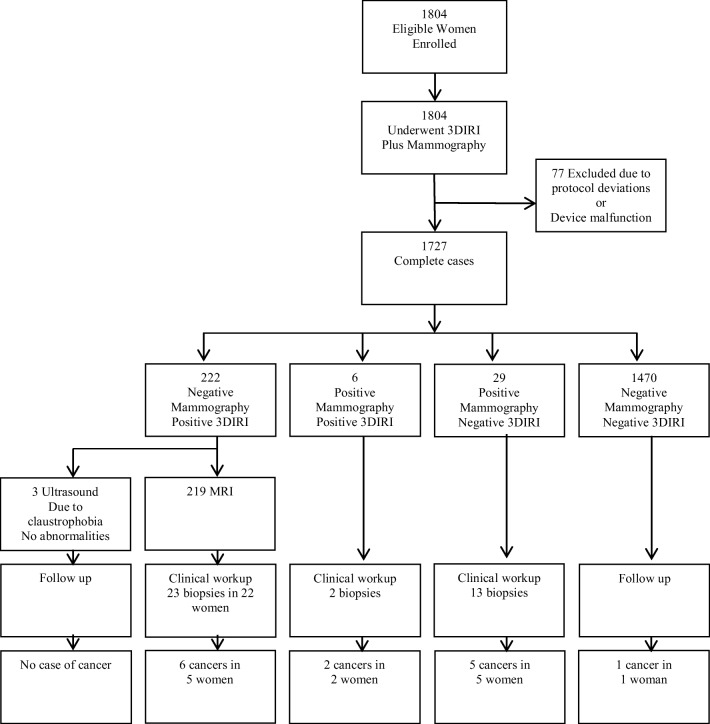
Outcome of screening with mammography and three-dimensional functional infrared imaging

Thirteen cancers were diagnosed in 12 women; imaging results and tumor characteristics are shown in Table 2. A total of 35 women (2%) were recalled on the basis of findings from the mammographic examination (Fig. 1). Of these women, 6 had a *positive* 3DIRI score and 29 a *negative* 3DIRI score. Fifteen of the 35 women had a breast biopsy, and a total of 7 cancers were detected in 7 women. Diagnostic yield of digital mammography was 7 of 1727 (0.41%; 95% CI 0.20–0.83%). Of these 7 women, 5 women had a negative 3DIRI score and the following histopathology: 15-mm invasive ductal carcinoma grade I, 5-mm invasive ductal carcinoma grade II, 5-mm invasive carcinoma grade III + 40-mm ductal carcinoma in situ grade III, 35-mm invasive ductal carcinoma grade III, 48-mm ductal carcinoma in situ grade II.

**Table 2:** Summary of 13 screen-detected cancers identified in 12 study women

Patient number	Age	Histopathological finding	Tumor size (mm)	Volumetric breast density^1^ (%)	Mammography screening result	3DIRI^2^ risk score	Distance to skin (mm)	Mammography breast thickness (mm)
1	60	Invasive ductal carcinoma grade I	15	18.2	Code 4	Negative (− 48.5)	15	67
2	55	Carcinoma in situ grade III	16	19.0	Code 3	Positive (4.9)	44	54
3	66	Carcinoma in situ grade III	43	44.5	Code 3	Positive (48.6)	65	42
4	56	Invasive ductal carcinoma grade I	7	18.9	Code 1	Positive (22.8)	16	80
5	72	Carcinoma in situ grade III	42	9.3	Code 1	Positive (12.1)	8	55
6	68	Invasive ductal carcinoma grade I	11	11.3	Code 1	Positive (41.9)	30	67
7	73	Carcinoma in situ grade I	7	32.3	Code 1	Positive (3.5)	7	63
8	74	Invasive ductal carcinoma grade II	5	7.5	Code 3	Negative (− 37.2)	18	59
9	59	Invasive ductal carcinoma grade III + cancer in situ grade III	5 + 40 mm	17.7	Code 5	Negative (− 26.6)	21	86
10	72	Invasive ductal carcinoma grade III	35	43.0	Code 5	Negative (− 22.4)	30	61
11	73	Carcinoma in situ grade II	48	5.3	Code 3	Negative (− 38.6)	13	63
12	70	Left: invasive ductal carcinoma grade II Right: invasive ductal carcinoma grade III	Multifocal Multifocal	9.6	Code 1	Positive (42.2)	Left: 23 Right: 22	Left: 55 Right: 55

^1^Mean volumetric breast density for right and left breasts

^2^Three-dimensional functional infrared imaging

**Fig. 2:**
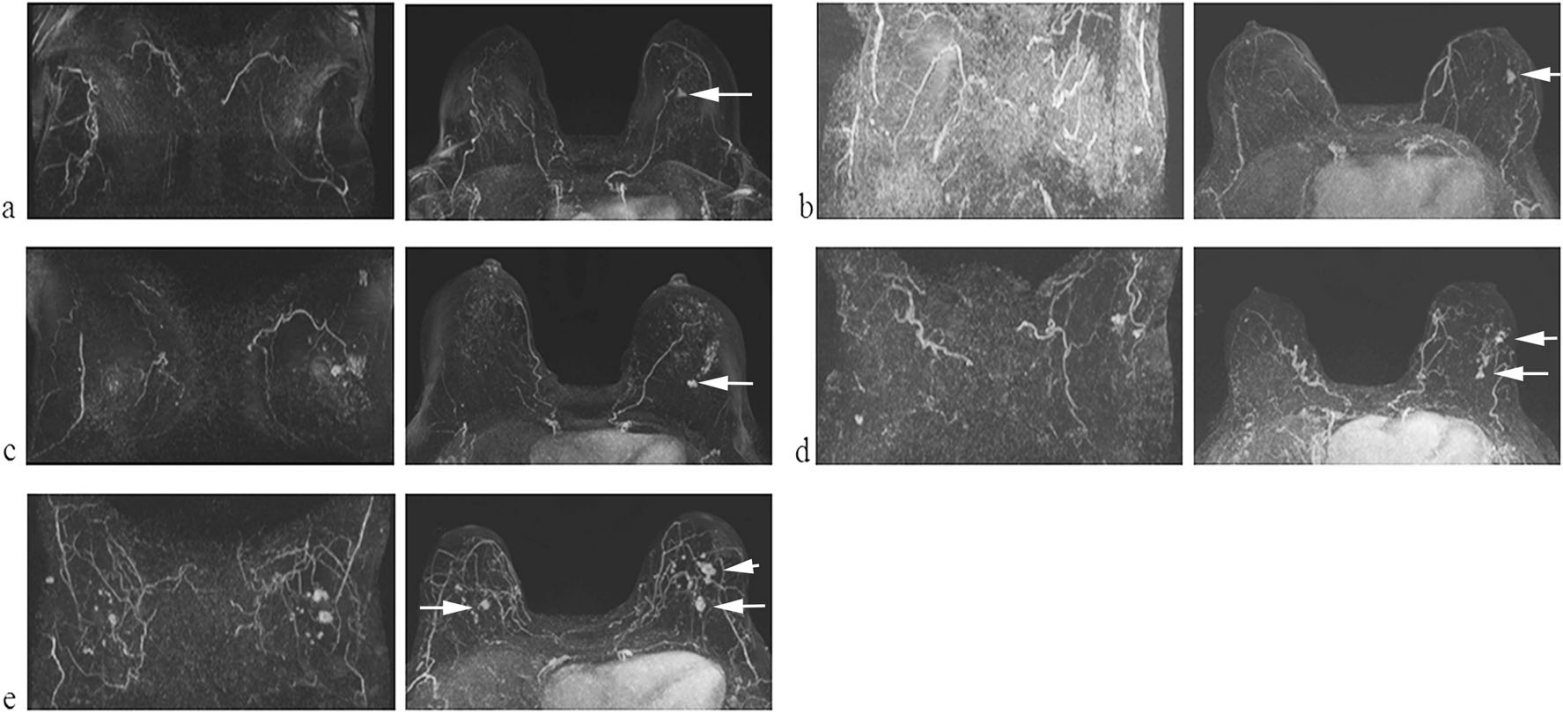
Maximum intensity projection of contrast-enhanced MRI of the 5 women with mammography-negative, 3DIRI-positive malignancies. The arrows indicate tumor. (**a**) 68-year-old woman with 11-mm invasive carcinoma in the left breast, (**b**) 73-year-old woman with 7-mm ductal cancer in situ in the left breast, (**c**) 56-year-old woman with 7-mm invasive ductal carcinoma in the left breast, (**d**) 72-year-old woman with 42-mm ductal cancer in situ in the left breast, (**e**) 70-year-old woman with bilateral multifocal ductal carcinoma

**Fig. 3:**
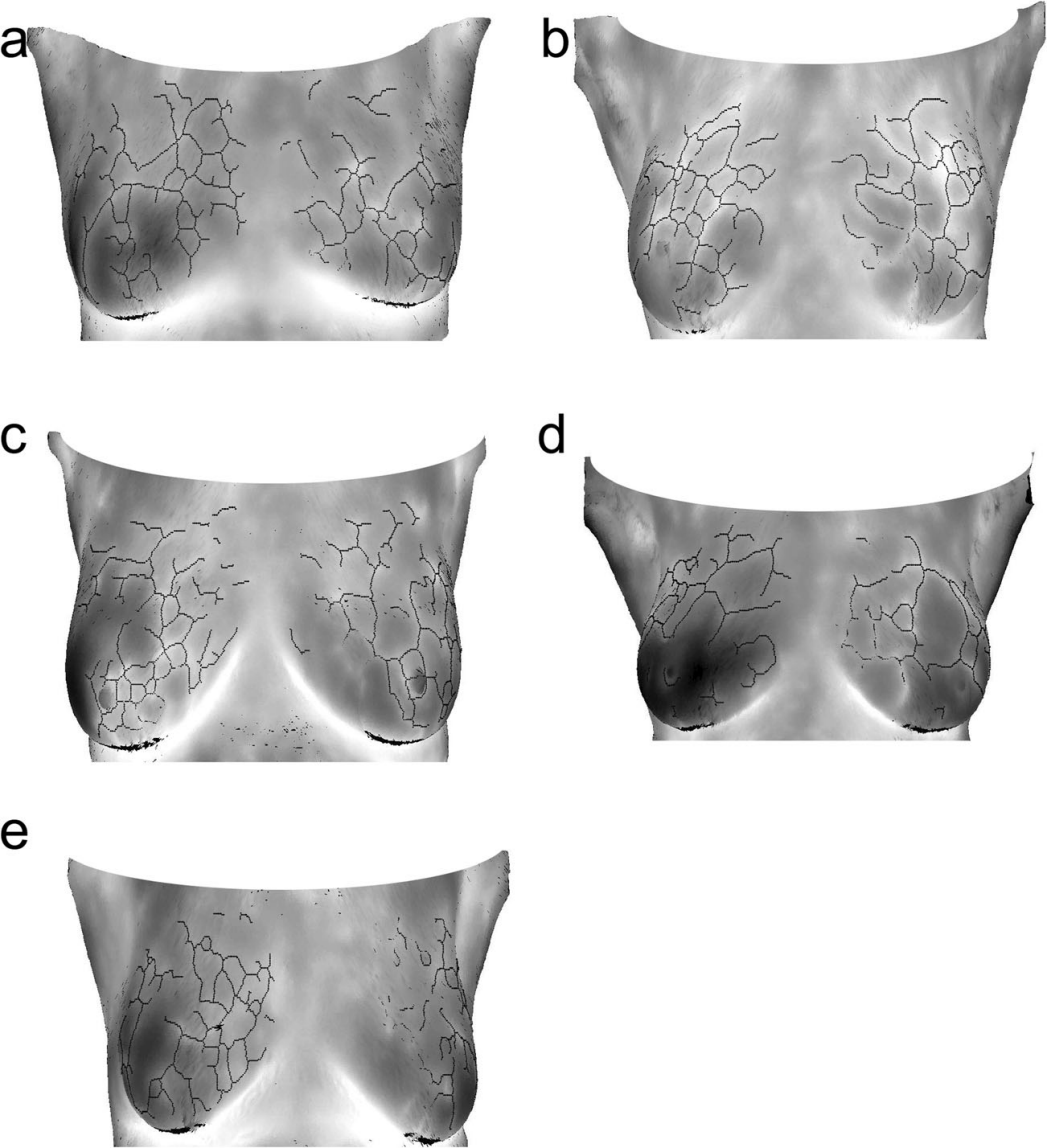
Projections of 3DIRI breast vascular maps of the 5 women with mammography-negative, 3DIRI-positive malignancies. Patient and tumor characteristics are described in the caption to Fig. 2

Of 1692 women with a negative mammogram, 222 women (13%) were recalled on the basis of *positive* 3DIRI score and referred to have an MRI. Of these 222 women, 219 (99%) underwent an MRI and 3 women underwent an ultrasound as they could not complete the MRI due to feelings of claustrophobia. Of 219 women who underwent MRI, 22 women (10%) had positive findings on the MRI and underwent additional workup and 6 cancers were detected in 5 women. We present the maximum intensity projection of contrast-enhanced MRI for these 5 women (Fig. 2) and breast vascular maps generated by the 3DIRI (Fig. 3) in the same order. Diagnostic yield of digital mammography with additional 3DIRI to select women for further examination was 12 of 1727 (0.69%; 95% CI 0.40–1.21%).

Of the 1470 women with a negative mammogram and negative 3DIRI, 1 woman was diagnosed with interval cancer with 1 year of follow-up.

According to the histopathological report, 8 out of 13 cancers (62%) were diagnosed as invasive and 5 (38%) as ductal carcinoma in situ. Tumor size and characteristics are reported in Table 2.

The incremental cancer detection rate associated with using 3DIRI to select women for MRI was 5 of 222 which translates to 22.5 additional cancers per 1000 women referred for MRI (95% CI 10–52).

### Positive predictive value of biopsy (PPV_3_)

One must keep in mind that biopsies performed when using 3DIRI to select for MRI are based on positive findings of MRI and not positive 3DIRI. Based on mammography alone, we identified 7 malignancies out of 15 biopsies giving a PPV_3_ of 0.47 (95% CI 0.25–0.70%).

Using 3DIRI to select for MRI, we identified 6 cancers in 23 biopsies giving a PPV_3_ of 0.26 (95% CI 0.13–0.46%).

Among the 25 biopsies that were negative for cancer, 8 were based on a positive mammography and 17 were based on a positive MRI. The biopsies based on a positive mammography showed 2 cases of benign breast tissue, 4 cases of fibroadenoma, and 2 cases of hyperplasia. The biopsies based on a positive MRI showed 3 cases of fibroadenoma, 2 cases of cystic fibroadenosis, 5 cases of benign fibroglandular tissue, 1 case of fibrosis, 3 cases of adenosis, and 3 cases of papilloma/papillomatosis.

### Estimates of diagnostic accuracy for the different modalities

Assuming an MRI, if performed, would not have detected any cancers among the women with a negative mammogram and negative 3DIRI at the time of screening, we calculated the diagnostic accuracy of mammography alone, 3DIRI alone, and the combination of mammography and 3DIRI (Table 3).

**Table 3:** Estimates of diagnostic accuracy for the different modalities

Modality	Number of women with cancer	Sensitivity	Specificity	Positive predictive value	Negative predictive value
Mammography	7	58% (7/12)	98% (1687/1715)	20% (7/35)	100% (1687/1692)
3DIRI	7	58% (7/12)	87% 1494/1715	3% (7/228)	100% (1494/1499)
Mammography + 3DIRI	12	100% 12/12	86% (1470/1715)	5% (12/257)	100% (1470/1470)

### Interval cancers

All study participants were followed up for breast cancer for 12 months by matching with the National Breast Cancer Registry, which has close to 100% ascertainment of breast cancers for women in the age group [25]. The register follow-up identified 1 participating woman with a breast cancer diagnosis, who had a negative mammogram and a negative 3DIRI at inclusion in our study. This woman was diagnosed with a 30-mm high-grade invasive cancer 315 days after being examined in our study. The rate of interval cancers in our region is approximately 2 per 1000 women, for a 2-year screening interval.

## Discussion

We used three-dimensional functional infrared imaging (3DIRI) to select 13% (*n* = 222) of the women with a negative screening mammography for additional screening with MRI and we detected cancers in 5 of the 222 women. This corresponds to an incremental cancer detection rate of 22.5 additional cancers per 1000 women referred for MRI.

The aim of our study was to estimate the incremental cancer detection rate achieved by adding 3DIRI to digital mammography. We did not aim to compare the incremental cancer detection rate to what might have been achieved using other methods, but we will discuss this as it is clinically important. As mentioned in the introduction, digital breast tomosynthesis is a mammography technique with increased sensitivity in women with dense breasts. Therefore, the added value of 3DIRI would likely be lower in a tomosynthesis-screened population. Another way of considering our results is comparison with the results of the study by Kuhl et al [26]. Although our study populations differ, our results are similar to their study in which MRI was added to screening mammography for randomly selected women, with an additional cancer detection of 22.6 per 1000 in the first MRI round. Another study by Chen et al showed that adding MRI to a screening population with dense breasts and a negative mammography gave additional cancer detection of 33 per 1000 examined women [27]. In our study, we chose to conduct MRI in cases with a positive 3DIRI and negative mammography, because of the high negative predictive value of MRI. However, in a population-based screening situation, the high number of MRI examinations would be problematic and costly. It would be much more acceptable if 3DIRI would triage for ultrasound. This in turn would reduce the incremental cancer detection rate as some of the cancers were only detected on MRI. Adding ultrasound to mammography increases cancer detection by approximately 30% [7, 8, 11]. The positive predictive values of biopsies after a positive ultrasound are reported to be in the range of 0.08–0.28 [7–9, 28]. The problem of ultrasound is that both handheld and automated methods are time consuming and heavily operator dependent. The advantage of 3DIRI is that the method is non-invasive, and the risk score is generated automatically and is immediately available to the radiologist.

As 3DIRI missed 5 mammography-detected cancers, we would not recommend replacing MRI with 3DIRI among women with a high lifetime risk of breast cancer. The results in this study suggest a relatively low correlation between mammography and 3DIRI. 3DIRI and mammography detect malignancies in different ways (evaluating changes in perfusion versus anatomical changes) and we do not expect perfect agreement between the two methods. While mammography detects changes in the anatomy/density of the breast tissue due to the presence of mass, 3DIRI detects changes in breast physiology. As such, some cancers with a large mass can be misclassified by 3DIRI since the changes in the physiology are below the detection threshold. The 3DIRI device detects difference in the venous vasculature close to the surface of the breasts. Two recent studies have demonstrated a strong correlation between asymmetric increased breast vascularity and cancer size. Kostopoulos et al [29] showed that automated breast vascular asymmetry assessment on MRI images of malignant tumors has an AUC of 84.2%. The authors concluded that small tumors may trigger the angiogenetic process that would affect the whole-breast vascularity. Bufi et al [30] found that asymmetric increased breast vascularity is not only a predictor of breast malignancy with high sensitivity, but can also predict complete pathologic response to neo-adjuvant chemotherapy. We therefore hypothesize that the detection of 3DIRI may be based on small changes in breast *vascularity*. Similar to the study by Sella et al [14], we found no evidence that the technology is less sensitive for large breasts or tumors deep in the breasts although we have few cancers in our study to draw any definitive conclusions. This is a major difference to previous infrared imaging techniques that relied only on the detection of hot spots caused by cancer. While this study was carried out by a prototype device, a newer implementation of the 3DIRI modality suggests a strong association between the 3DIRI scores and hormonal status of the women [31]. In our study, 3DIRI was performed without the menstrual cycle taken into consideration. This may have impacted on the number of false positives. Women are now recommended to undergo 3DIRI during their follicular phase of their menstrual cycle (similar to MRI) in order to avoid false-positive 3DIRI scores. Further analysis and improvement of the 3DIRI modality is still needed to address the concern of misclassification.

The current study has some limitations. First, the radiologists working with our study were experienced in reading digital mammography but relatively inexperienced with breast MRI compared, for example, to the radiologists working with other MRI studies we have cited. It is possible we may have failed to identify additional cancers on some MRI images, although so far no interval cancers have been diagnosed among these women who underwent MRI. Second, the incremental cancer detection rate is based on a small number of cancers and is therefore too small to draw conclusions. We presented estimates of diagnostic accuracy for the different modalities, but these are based on strong assumptions and should be interpreted with caution. We did not perform an MRI for women with a negative mammography and negative 3DIRI. The estimates in Table 3 are based on the assumption that an MRI would not have detected any cancers among these 1470 women at the time of screening. The occurrence of one interval cancer suggests this assumption may not be valid. 3DIRI is intended as an adjunct to mammography so the estimates of diagnostic accuracy for 3DIRI alone do not represent how it performs in its intended purpose. On the other hand, the estimated sensitivity for mammography plus 3DIRI is not representative since it has been estimated under the assumption the combined modality had perfect sensitivity. Our study was not designed to compare the diagnostic accuracy of various modalities, but the comparison is of potential interest so we have provided speculative estimates as a basis for comparisons.

To this end, we propose to perform a multicase multireader study on all cancer and some negative cases in this cohort to determine if the incremental cancer yield is statistically significant and it could further assess if 3DIRI can be utilized as an adjunct to mammography overcoming the ‘automatic recall’ carried out in our study. Also, a randomized control study in which participants are randomized to either 3DIRI or an alternative adjunct screening procedure (e.g., ultrasound) can provide more information about the clinical benefit and cost-effectiveness of 3DIRI.

## Conclusions

Selecting asymptomatic women for MRI based on screening 3DIRI risk score will result in increased cancer detection, but at the expense of additional false positives and considerably lower positive predictive value of the combined examinations. Further studies that use a consistent standard for validation are necessary to correctly evaluate the accuracy of 3DIRI.

## Electronic supplementary material

ESM 1 (DOCX 1229 kb)

## Abbreviations

3DIRI: Three-dimensional functional infrared imaging
BIRADS: Breast imaging reporting and data system
