# Supplementary material for: Does three-dimensional functional infrared imaging improve breast cancer detection based on digital mammography in women with dense breasts?
Source: Eur Radiol. 2019 May 21;29(11):6227–35. doi: 10.1007/s00330-019-06248-y (PMC6795638; doi:10.1007/s00330-019-06248-y)
Supplement: Supplementary file 1 — (DOCX 1229 kb) [file 330_2019_6248_MOESM1_ESM.docx]

**Appendix 1**

**3DIRI imaging**

The 3DIRI device is composed of two identical optical heads placed symmetrically with regard to a vertical mid-plane through the subject sitting position. Each optical head comprises three main elements: a visible domain digital camera, an infrared camera, and a digital LED projector. The corresponding optical axes (shown in green, red and blue, respectively) are set to converge in the vertical mid-plane. This method requires calibration of each imaging/projecting element by estimating the projective matrix that maps any point in 3-D space into a 2-D pixel (Appendix figure 1).

**Appendix figure 1**. Schematic diagram showing optical paths of imaging components

The visible domain 3-D is reconstructed using a classical structured-light method wherein Gray coded binary patterns are sequentially projected on the breast of the subject and imaged by the visible domain camera (Appendix figure 2).


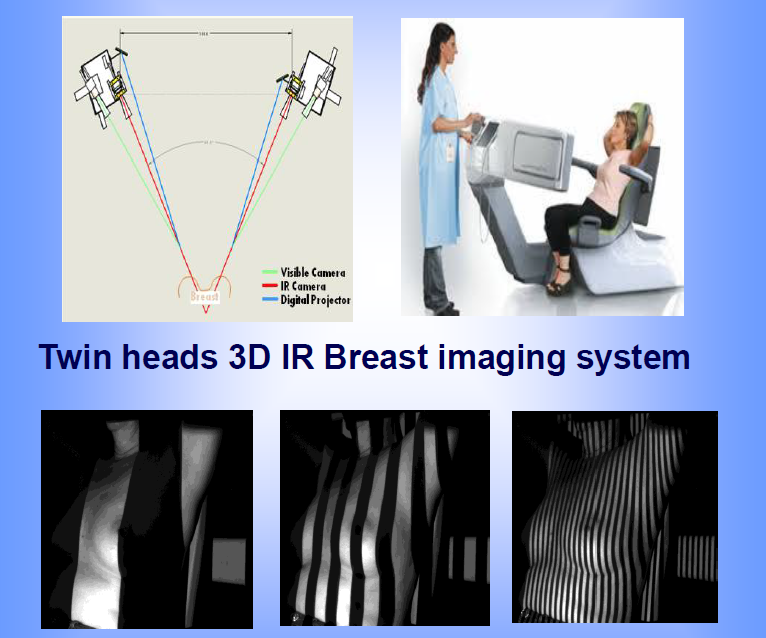

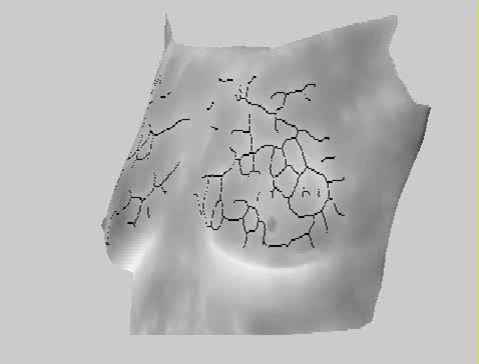


**Appendix figure 2**. Gray-coded patterns projected on the women for structured-light imaging

The resulting left and right point clouds are merged into a single 3-D (2.5D) bust. Eventually, the images from both infrared cameras are back projected onto the point cloud to generate the infrared-textured bust surface (Appendix figure 3).


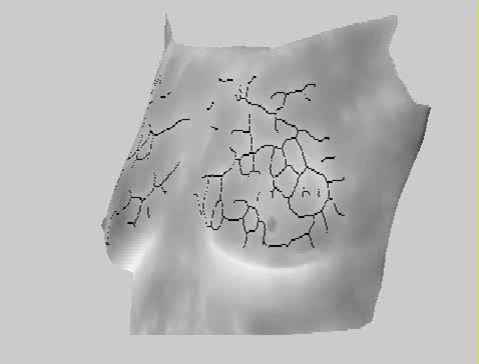


**Appendix figure 3**. Segmented vascular map

Prior to imaging, the patient will be summoned to the examination room. She will be instructed to remove her upper garments including any necklaces, nipple piercing or any other obstructions and to move long hair from the front of the body. The patient will then be dressed with a loose gown and be seated in the Patient Support for 15 minutes of temperature stabilization while her hands are placed on the supporting handles.

After 15 minutes, the patient will be instructed to take off her gown and face the Optic Heads in order for the operator to perform alignment of the Optic Heads.

The imaging procedure takes approximately 6.15 minutes and includes a total of seven sequences. Continuous infrared imaging is acquired for a period of 5 minutes. Two minutes after onset of the infrared recording, a stress test is induced by having the patient wears cold gloves (5 °C), which are then removed after 1 minute. This is performed for the purpose of generating vasoconstriction in the breast parenchyma. The entire imaging session lasts approximately 20 minutes.

Following the scan, a series of 3-D infrared image maps are generated. The image maps are automatically segmented based on the 3-D breast digital model, in order to define the region of interest, which includes the entire breast, further specifying the area for evaluation. For each image set, a 3-D vascular map is generated. Analysis for the presence of tumor is based on multiple computerized algorithms which test the likelihood of malignancy. The 3DIRI device assesses differences in morphology, structure, contralateral asymmetry and temporal changes between contralateral vascular maps. Vascular maps are derived from the 3-D infrared imaging data and are further analyzed for the likelihood of malignancy. Various descriptors have been validated using a cohort of women with histology confirmed breast cancer imaged by both MRI and 3DIRI, and a cohort of healthy women, also imaged by both MRI and 3DIRI with no cancer for one year. Histology confirmed cancer cases in this cohort were selected based on increased vascularity (determined subjectively by a radiologist from MRI images). Descriptors were optimized based on area under the curve (AUC) of Receiver Characteristic Operation (ROC).
